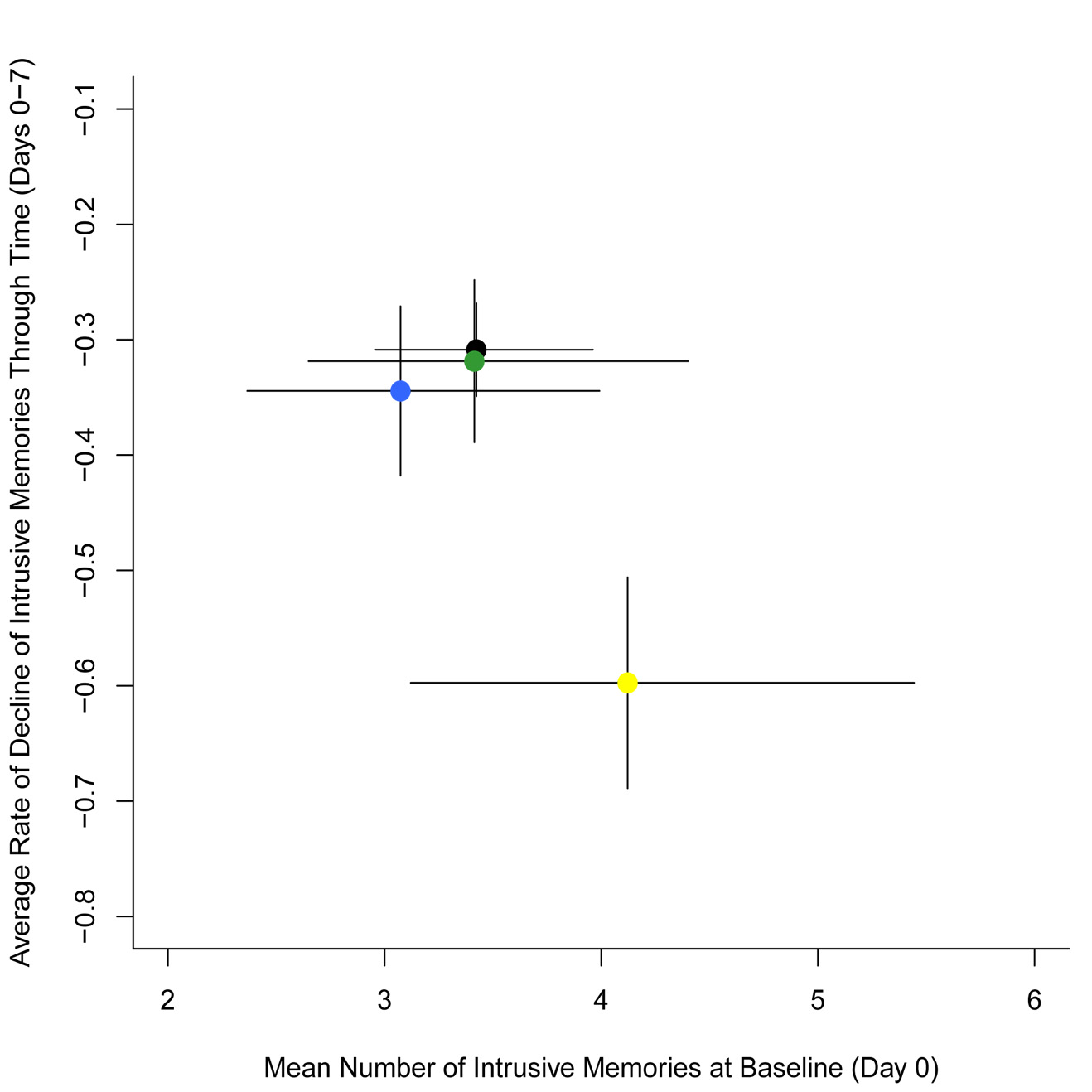# Computer Game Play Reduces Intrusive Memories of Experimental Trauma via Reconsolidation-Update Mechanisms

**DOI:** 10.1177/0956797615583071

**Published:** 2015-08

**Authors:** Ella L. James, Michael B. Bonsall, Laura Hoppitt, Elizabeth M. Tunbridge, John R. Geddes, Amy L. Milton, Emily A. Holmes

**Affiliations:** 1 Medical Research Council Cognition and Brain Sciences Unit, Cambridge, United Kingdom; 2 Department of Psychiatry, University of Oxford; 3 Department of Zoology, University of Oxford; 4 St Peter’s College, University of Oxford; 5 Department of Psychology, University of Cambridge; 6 Department for Clinical Neuroscience, Karolinska Institutet

**Keywords:** intrusive memory, intrusions, reconsolidation, computer game, involuntary memory, trauma film, mental imagery, emotion, open data, open materials

## Abstract

Memory of a traumatic event becomes consolidated within hours. Intrusive memories can then flash back repeatedly into the mind’s eye and cause distress. We investigated whether reconsolidation—the process during which memories become malleable when recalled—can be blocked using a cognitive task and whether such an approach can reduce these unbidden intrusions. We predicted that reconsolidation of a reactivated visual memory of experimental trauma could be disrupted by engaging in a visuospatial task that would compete for visual working memory resources. We showed that intrusive memories were virtually abolished by playing the computer game *Tetris* following a memory-reactivation task 24 hr after initial exposure to experimental trauma. Furthermore, both memory reactivation and playing *Tetris* were required to reduce subsequent intrusions (Experiment 2), consistent with reconsolidation-update mechanisms. A simple, noninvasive cognitive-task procedure administered after emotional memory has already consolidated (i.e., > 24 hours after exposure to experimental trauma) may prevent the recurrence of intrusive memories of those emotional events.

Psychological trauma is prevalent around the world (World Health Organization, 2013), from terrorist attacks to motor vehicle accidents. Most people will experience a traumatic event during their life. Some will develop “recurrent, involuntary and intrusive distressing memories of the traumatic event(s)” (*Diagnostic and Statistical Manual of Mental Disorders*, 5th ed., or *DSM–5*; American Psychiatric Association, or APA, 2013, p. 271). Intrusive memories are a hallmark of acute stress disorder and posttraumatic stress disorder (PTSD; APA, 2013), but understanding emotional and intrusive memory has broader relevance beyond trauma—involuntary images of various emotional autobiographical events are common in daily life (Bernsten, 2010). Ways to modulate the persistence of intrusive memories are little understood.

Effective mental-health interventions soon after trauma are lacking (Roberts, Kitchiner, Kenardy, & Bisson, 2010). Disaster-response aid can be mobilized 24 to 48 hr after an event (American Red Cross, 2010), but within the first 6 hr, emotional memories are already consolidated and change resistant (McGaugh, 2000). Procedures that could alter a consolidated trauma memory are critical for reducing posttraumatic symptoms. It is time to profit from advances in the science of memory to devise innovative psychological treatments (Holmes, Craske, & Graybiel, 2014).

People may wish to forget traumatic memories, but counterintuitively, they may benefit from recalling them under certain conditions—those which render them less intrusive. Reconsolidation is the process whereby reactivation of a previously consolidated memory renders it malleable, and restabilization is required for the memory to persist (Misanin, Miller, & Lewis, 1968; Nader, Schafe, & LeDoux, 2000). Memory changes when an intervention disrupts or enhances restabilization. For reconsolidation to occur, the memory must be reactivated via a retrieval cue (Merlo, Milton, Goozee, Theobald, & Everitt, 2014). While it is malleable, the reactivated memory can be updated—weakened or strengthened (or unchanged)—for example, by pharmacological agents. Intracranially delivered protein-synthesis inhibitors block reconsolidation and weaken fear memory in animals (Nader et al., 2000) but are toxic in humans. Studies in humans have used the beta-blocker propranolol to reduce physiological responses to conditioned fear cues in healthy volunteers (Kindt, Soeter, & Vervliet, 2009). Clinical findings are mixed, and translation to PTSD requires caution (Lonergan, Olivera-Figueroa, Pitman, & Brunet, 2013).

Various nonpharmacological techniques have been employed in experimental trials with humans. Electroconvulsive therapy has been used to disrupt reconsolidation of episodic memories (Kroes et al., 2014), although this sort of therapy is distressing. Memory extinction during reconsolidation has been used to reduce conditioned fear to picture cues (e.g., blue squares) in healthy participants with long-lasting effects (Schiller et al., 2010) and concomitant changes in amygdala activity (Ågren et al., 2012). However, it remains to be shown whether the frequency of intrusive memories of an experimental analogue of traumatic events can be reduced by targeting reconsolidation. This is important because intrusive memory (rather than deliberately recalled episodic memory) is central to clinical posttraumatic distress. Furthermore, it remains to be demonstrated that simple, noninvasive cognitive techniques might reduce intrusions. Such techniques could be readily administered in the aftermath of real trauma.

We predicted that engaging in a visuospatial task during memory reconsolidation would compete for working memory resources with visual imagery and interfere with the reconsolidation of intrusive memories. There are dual-task experiments indicating that when similar cognitive tasks compete for shared resources, they interfere with each other and thereby impede memory processing; for example, a visuospatial pattern-tapping task interfered with holding a visual mental image in mind (rendering it less vivid and emotional), whereas counting aloud did not (Baddeley & Andrade, 2000). Conversely, counting aloud had a detrimental effect on an auditory memory, whereas visuospatial tapping did not. Such a dual-task capacity limitation provides an advantage by which to limit resources allocated to maladaptive forms of cognition. Intrusive memories of trauma consist of mental images such as visual scenes from the event (Brewin, 2014), for example, the sight of a red car moments before a crash. Therefore, a visuospatial task performed when memory is labile (during consolidation or reconsolidation) should interfere with visual memory storage (as well as restorage) and reduce subsequent intrusions.

Concurrent tasks may interfere with each other, and such interference can influence their consolidation into memory. A task performed after an event may retroactively interfere with memory for that event (Wixted, 2004). Consistent with this hypothesis, previous studies have shown that visuospatial tasks, such as the computer game *Tetris* (Green & Bavelier, 2003), performed during (Bourne, Frasquilho, Roth, & Holmes, 2010; Holmes, Brewin, & Hennessy, 2004) or soon after (Deeprose, Zhang, Dejong, Dalgleish, & Holmes, 2012; Holmes, James, Coode-Bate, & Deeprose, 2009; Holmes, James, Kilford, & Deeprose, 2010) experimental trauma result in fewer subsequent intrusions than when no task or a verbal-based task is performed (Bourne et al., 2010; Deeprose et al., 2012). Recently, it has been argued that any dual task that sufficiently taxes working memory (rather than its modality specificity) should influence memory emotionality (van den Hout & Engelhard, 2012; although see Brewin, 2014). Because our interest in the present study was in visual imagery, we adopted the conservative approach of selecting a taxing *visuospatial* task to create a capacity limitation. Critically, our main question was whether intrusions can be disrupted once consolidated. The current experiments tested whether we could ameliorate the frequency of intrusions of an already consolidated memory for a traumatic film: To do this, we asked participants to play the computer game *Tetris* after memory reactivation performed in the time window for disrupting reconsolidation.

We used the traumatic-film paradigm because it is a well-established prospective experimental tool for investigating intrusive-memory development (Holmes & Bourne, 2008; Horowitz, 1969). In this paradigm, which has been used to study intrusions in behavioral studies (e.g., Deeprose et al., 2012; Hagenaars & Arntz, 2012; Holmes et al., 2009; Holmes et al., 2010) and neuroimaging studies (Bourne, Mackay, & Holmes, 2013), participants are shown short films containing scenes depicting traumatic events. Such films reliably induce intrusions over the following week. Notably, a correlational study found that repeatedly viewing media related to the Boston Marathon bombing (6 hr or more daily) was associated with higher acute stress symptoms than direct exposure to that event (Holman, Garfin, & Silver, 2014). Prospective longitudinal data suggest that TV-related exposure to the events of September 11, 2001, was associated with posttraumatic stress symptoms over the following 3 years (Silver et al., 2013).

We hypothesized that 24 hr after experimental trauma-film exposure, a group that completed a reactivation task for memory of the film (to initiate reconsolidation) followed by *Tetris* game play would have a lower frequency of subsequent intrusions, compared with control groups that completed only one or none of those tasks.

## Experiment 1

In Experiment 1, we compared two experimental groups, predicting that a group that completed a memory-reactivation task plus *Tetris* game play would show a lower frequency of intrusive memories of a traumatic film, compared with a control group given no tasks. Intrusive memories were assessed in daily life (diary methodology; e.g., Holmes et al., 2009; Holmes et al., 2010) and through a laboratory task (intrusion-provocation task, or IPT). On Day 0, participants completed baseline questionnaires assessing past trauma history, trait anxiety, and depressive symptoms, then viewed the trauma film. Then they recorded, in a diary, intrusions of the film over the next 24 hr. On Day 1, participants returned to the laboratory and were randomly assigned to two groups: The reactivation-plus-*Tetris* group (*n* = 26) completed a memory-reactivation task—presentation of 11 film stills followed by a filler task for 10 min (Ågren et al., 2012; Schiller et al., 2010)—and then played *Tetris* for 12 min. The control group (*n* = 26) was neither given the memory-reactivation task nor played *Tetris*; rather, after the 10-min filler task, they had a 12-min break in which there was no task. Subsequently, both groups continued to record intrusive memories for 7 days (Days 1–7). The diary allowed investigation of the day-by-day time course of memory intrusions and their overall frequency. On Day 7, participants returned to the laboratory and recorded intrusions on a convergent measure (IPT). Finally, both groups completed recognition memory tests to confirm equivalent recognition for film content (Brewin, 2014; Holmes et al., 2009; Holmes et al., 2010).

### Method

#### Participants

Fifty-two participants (31 females, 21 males; age range = 18–51 years) were recruited from two local university campuses and from the general public via advertisements in an online newspaper and in the community. Sixty-five percent of participants were students, 21% were employed, and 14% were unemployed. Participants described their ethnicity as 52% White British, 19% White other, 12% Asian Indian, 4% Chinese, 4% Asian Pakistani, 2% Black African, 2% Black American, 2% Latin American, 2% White American, and 2% White Asian. For ethical considerations, the recruitment material gave information about the nature of the film, specifically, that it contained scenes of a traumatic or potentially distressing nature. All participants provided written informed consent prior to testing, were reminded that they could end the experiment at any point, and were reimbursed for their participation. Participants were required to complete all three lab sessions (on Days 0, 1, and 7) and keep the diary in between sessions. No participants had been involved previously in related studies. Ethical approval was obtained from the University of Oxford Central University Research Ethics Committee (reference number: MSD/IDREC/C1/2010/104).

#### Tasks and measures

##### Trauma film

The 12-min trauma film consisted of 11 different scenes involving actual or threatened death, as well as serious injury; the film functioned as an experimental analogue of viewing a traumatic event in real life (APA, 2013). Scenes contained different types of context; examples include a young girl hit by a car with blood dripping out of her ear, a man drowning in the sea, and a van hitting a teenage boy while he was using his mobile phone crossing the road. This film footage has been used in previous studies to evoke intrusive memories (e.g., Holmes et al., 2009; Holmes et al., 2010). The film was projected on a 100-cm × 133-cm screen using an NEC LT25 projector. Viewing distance was approximately 175 cm.

##### Memory-reactivation task

The memory-reactivation task had two components: (a) presentation of still film images then (b) a 10-min break with a standardized filler task. For the first part of the task, 11 static visual images, one from each of the scenes in the film were presented once each on a black background for 2 s using PowerPoint. Examples included a young girl sitting up at the side of the road (just before the moment in the film clip when she is hit by a car), a man from the torso up striding through the sea (just before the moment in the film where he drowns), and the face and shoulders of a teenage boy smiling at the camera (just before he replies to a text message while being hit by a van). Images were taken from a moment just prior to the worst part of a given scene (i.e., those which typically yield intrusive memories; cf. Michael & Ehlers, 2007).

The images were intended as reminder cues for the trauma film (though there was no explicit instruction to this effect, and there was no explicit instruction to deliberately recall the film). Images were projected with the same equipment and procedure as during film viewing. The images were presented in the same fixed order as the scenes had occurred in the film. Second, as part of the task, and to allow time for memory reconsolidation processes to be initiated, we provided a 10-min interval after the presentation of the images but before the blockade (*Tetris* game play). Such a break is used in both animal (Nader et al., 2000) and human reconsolidation literature (Ågren et al., 2012; Schiller et al., 2010). The break contained a standardized music filler task, during which excerpts of classical music were rated for pleasantness.

##### Tetris

In the PC game *Tetris* (Version 1.2.1; Blue Planet Software, 2007), seven differently shaped and brightly colored geometric blocks (e.g., a blue rectangle, an orange T shape) fall from the top to the bottom of the playing screen in a random sequence one at a time. Using the keyboard arrow keys, players can move the blocks left or right, rotate them 90°, or accelerate them down as they fall to the bottom of the playing screen. The aim is to create complete horizontal lines across the bottom of the playing area using the blocks. Each time a full horizontal line is created, it disappears, and the participant is awarded points. In the current experiment, participants were reminded to focus on the three blocks due to fall after the one that they were currently manipulating (these blocks were displayed in a preview to the right of the playing screen). To encourage mental rotation, we asked participants to work out in their “mind’s eye” where best to place these blocks in order to create the horizontal lines and be awarded points. This version of *Tetris* was set to “Marathon” mode and played with the sound off. Each participant’s cumulative *Tetris* score was noted and performance self-ratings taken (see the Supplemental Material available online for details).

##### Intrusion diary

Participants were given a pen-and-paper diary to record any intrusive memories of the film content for the first 24 hr (Day 0) and again for Days 1 to 7 (Holmes et al., 2004; Holmes et al., 2009). Participants were advised (both verbally and by written instructions in the diary) that intrusive memories were defined as scenes of the film that appeared spontaneously and unbidden in their mind. They were not to include memories that they deliberately recalled. Participants were also given instructions about the form of intrusive memories, that is mental images (e.g., “in the form of pictures in your mind’s eye”) rather than solely verbal thoughts in the form of words or phrases (only those with image-based content were scored). They were asked to describe the content of each of their intrusions in the diary (e.g., a silver car crushing couple against a wall) so that the experimenter could later confirm whether or not the intrusion related to the film. Each day of the diary was labeled and split into three sections (morning, afternoon, and evening), and participants were asked to mark in a box in the appropriate section when they experienced an intrusive memory (or to indicate that they had not), and then to write the content of the intrusion overleaf. They were asked to record all intrusions immediately and to set aside regular time slots to check that their diary was up to date each day. If participants had experienced no intrusions during any time period, they were asked to enter zero in the diary.

##### Intrusion-provocation task (IPT)

Stimuli for the IPT consisted of 11 blurred static visual images created using GIMP (Version 2.1.1; Free Software Foundation, 2010) software (Gaussian Blur set at 2.0). There was one image from each scene of the trauma film. The images were presented for 2 s each on a 17-in. color monitor on a white background; participants sat at a viewing distance of approximately 75 cm. Images were presented in a fixed random order. Immediately afterwards for the next 2 min, participants recorded any intrusive memories triggered of the film by pressing a button. Intrusive memories were defined as in the diary. The total frequency yielded the IPT intrusion score.

##### Recognition memory tests

The verbal recognition memory test comprised 32 true/false written statements relating to the 11 scenes in the trauma film. Examples included “Scene 1: The little girl has blood coming from one of her nostrils” and “Scene 7: A man swims out to retrieve an inflatable lilo [beach air mattress]” (Holmes et al., 2009; Holmes et al., 2010).

The visual recognition memory test consisted of 22 static visual images—11 were taken from throughout the film (1 per scene, different images from those used in the IPT), and 11 were unviewed images presented as filler. Images were presented individually for 5 s each. For both visual and verbal recognition memory tests, participants indicated yes or no (on paper) as to whether or not they recognized the image (visual recognition memory test) or written statement (verbal recognition memory test) as being from the film watched 8 days earlier.

##### Self-report questionnaires

Prior trauma history was reported using the Traumatic Experience Questionnaire (TEQ), a 12-item checklist adapted from the Criterion A list of the Posttraumatic Diagnostic Scale (Foa, 1995), as per previous studies (e.g., Holmes et al., 2010). Participants indicated whether or not they had experienced or witnessed each of a series of traumatic events. “Yes” answers were summed and could range from 0 (*no traumatic event*) to 12 (*each and every type of traumatic event experienced or witnessed*).

Depressive symptomatology was measured using the second edition of the Beck Depression Inventory (BDI-II; Beck, Steer, & Brown, 1996). The BDI-II is a 21-item self-report measure. Each item is measured on a scale from 0 to 3, with total scores ranging from 0 to 63; higher scores indicate greater levels of depression. The BDI-II has high internal consistency in clinical outpatients (α = .92) and student samples (α = .93; Beck et al., 1996).

Trait anxiety was measured using the trait scale of the Spielberger State-Trait Anxiety Inventory (STAI-T; Spielberger, Gorsuch, Lushene, Vagg, & Jacobs, 1983). The STAI-T is a 20-item self-report measure. Each item is rated on a 4-point scale, with scores range from 20 to 80; higher scores represent greater levels of trait anxiety.

Participants also completed the Intrusion subscale of the Impact of Event Scale—Revised (IES-R; Weiss & Marmer, 1997) as an additional exploratory measure. Participants responded to eight items in reference to the film (e.g., “Pictures about *the film* popped into my mind”) by indicating how distressing each item had been “during the past 7 days *with respect to the film you watched last week.*” Items were rated on a 5-point Likert scale from 0 (*not at all*) to 4 (*extremely*).

#### Procedure

The experiment involved three laboratory sessions as well as the completion of a pen-and-paper diary at home to record the daily frequency of intrusive memories (both over 24 hr and then for an additional 7 days). The first laboratory session (Day 0) consisted of film viewing, and in the second session (Day 1), participants were assigned to experimental groups. The two sessions were conducted 24 hr apart to within 2 hr of the original testing time. The third experimental session occurred 7 days later, also to within 2 hr of the original testing time (Day 7).

On Day 0, participants provided written informed consent then completed information on their age, gender, occupation, and ethnicity, as well as the BDI-II, STAI-T, and TEQ. All participants were then asked to practice playing *Tetris* for 3 min on a 17-in. color monitor with the sound off.

Next, participants watched the trauma film alone in a darkened room. They were asked to pay close attention and to “imagine you are there, as a bystander at the scene.” Participants rated how sad, hopeless, depressed, fearful, horrified, and anxious they felt “right at this very moment” on six visual-analogue scales given both before and after the film. Ratings were made on scales from “*not at all*” to “*extremely*” (yielding a composite score; cf. Holmes et al., 2010). After film viewing, participants were also asked to rate “how distressing did you find the film you just watched?” on a scale from 0 (“*not at all*”) to 10 (“*extremely*”) and “how much attention did you pay to the film you just watched?” Participants were asked to note down any intrusive memories they experienced of the film in the diary for the next 24 hr. Finally, they were told to return the diary 24 hr later at their next laboratory session (on Day 1).

Immediately prior to their return to the laboratory for the second session, participants were randomly allocated to two groups (reactivation-plus-*Tetris* group: *n* = 26; no-task control group: *n* = 26). Participants in the reactivation-plus-*Tetris* group were given the memory-reactivation task, before which they were told, “I am about to show you a selection of pictures from the film you watched yesterday. I would like you to sit still and pay close attention. Do not look away, and really immerse yourself in viewing the pictures.” This was followed by the 10-min music filler task, and then participants played *Tetris* for 12 min. Participants in the control condition completed only the filler task (also for 10 min) and were then asked to sit quietly for 12 min (the period of time equivalent to *Tetris* game play) for which they were told, “There will now be a short break. Please stay seated and do not talk to the experimenter during this period. You can think about anything, with no restrictions.”

All participants were then reminded of the instructions for keeping the diary and were asked to keep the diary daily for a further 7 days. Seven days later (on Day 7), participants returned to the laboratory for the third and final session with their completed diary. In this session, they completed the IPT. They also rated how accurately they had completed their diary (diary compliance) from 1 (*not at all accurately*) to 10 (*extremely accurately*) and completed the verbal and visual recognition memory tests for trauma-film content, demand ratings, and the IES-R Intrusion subscale. Finally, participants were thanked, debriefed, and reimbursed for taking part.

#### Statistical analysis

On the basis of a between-groups effect size of *d* = 0.91 found by Holmes et al. (2009), we assumed a more conservative but still large effect size of 0.8 in moving from a memory consolidation to a reconsolidation time window. A sample size of 26 per group was required to ensure 80% power to detect this difference at the 5% significance level. We continued recruiting until we had reached the number of participants required based on our sample size. We used *t* tests for between-groups comparisons of intrusive memory frequency across the first 24 hr (prior to intervention), intrusive memory frequency across Days 1 to 7 (postintervention), IPT intrusion score, score on the Intrusion subscale of the IES-R, recognition memory scores, TEQ score, depression, anxiety, age, attention to the film, and diary compliance. Gender was analyzed between groups using a chi-square test. To assess mood deterioration resulting from viewing the trauma film, we conducted a two-way repeated measures analysis of variance (ANOVA) with main factors of time (pre- vs. postfilm) and group (reactivation-plus-*Tetris* vs. control). Two-tailed tests and an alpha level of .05 were used for all statistical comparisons. Time-series analyses were undertaken in *R.* (Analyses of the Intrusion subscale of the IES-R, the TEQ, depression, anxiety, age, film-related distress, attention to the film, diary compliance, demand, gender, and mood deterioration over film viewing are presented in the Supplemental Material.)

To investigate the time-course of intrusions, we ran a repeated measures analysis of covariance (ANCOVA) followed by nonlinear time-series analysis. Counts of the number of intrusive memories for each participant (*Y*) through time (*t*) were fitted with a generalized additive model (Hastie & Tibshirani, 1990):

Y(t)~Poisson(u(t))

log(u(t))=intercept+s(t,4),

where *u* is a random variable of time and *s*(*t*, 4) is the smoother with four effective degrees of freedom. The nonparametric form of the line was plotted on the intrusive-memory observation data, and expected Poisson distributions at Day 0, Day 1, and Day 2 were generated.

### Results

Groups were equivalent at baseline for age and gender, as well as self-report-questionnaire scores for trait anxiety, depression, and trauma history. Mood deterioration during film viewing, self-reported postfilm distress, attention to the film, demand ratings, and diary compliance also did not differ significantly between groups (see the Supplemental Material).

#### Intrusive memories preintervention

**Fig. 1.**
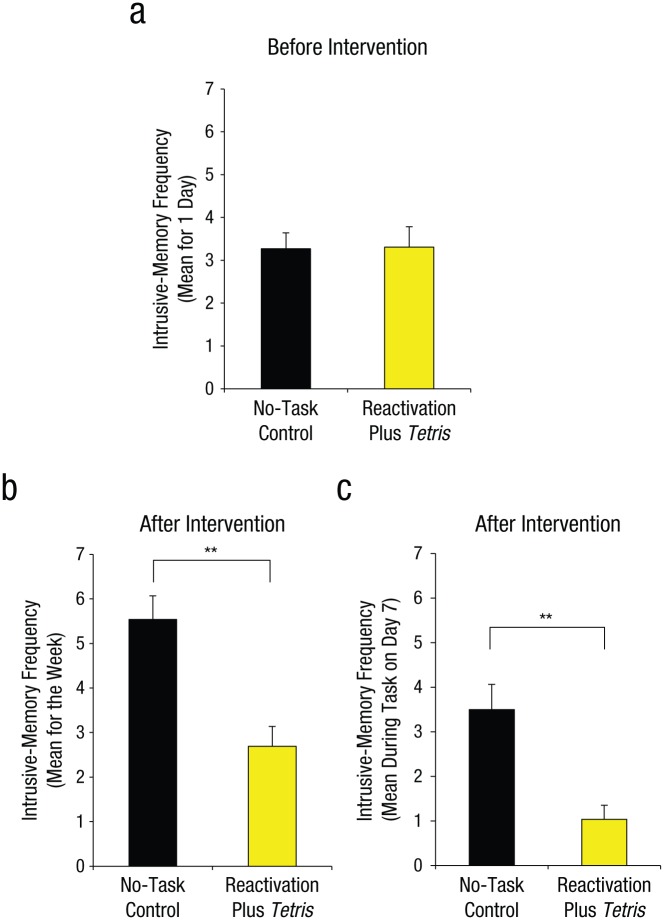
Results from Experiment 1: mean number of intrusive memories recorded in the diary during the first 24 hr following viewing of the experimental trauma film (i.e., preintervention; a), mean number of intrusive memories recorded in the diary totaled over the 7-day period after the intervention (b), and mean score on the intrusion-provocation task on Day 7 (c). In each graph, results are shown separately for the two groups. Asterisks indicate a significant difference between groups (**p < .001). Error bars represent +1 *SEM*.

As expected, prior to the intervention (at baseline: Day 0), we confirmed that the two groups experienced a similar number of intrusive memories of the film in daily life, *t*(50) = 0.06, *p* = .95 (Fig. 1a).

#### Intrusive memories postintervention

Critically and as predicted, after the intervention (Days 1–7; Fig. 1b), participants in the reactivation-plus-*Tetris* group overall had fewer intrusive memories in daily life than did those in the control group, *t*(50) = 4.11, *p* < .001, *d* = 1.14. Furthermore, a similar pattern was seen on a convergent measure—the frequency of IPT intrusions assessed on Day 7 in the laboratory, *t*(50) = 3.80, *p* < .001, *d* = 1.05 (Fig. 1c).

#### Time course of intrusions

**Fig. 2.**
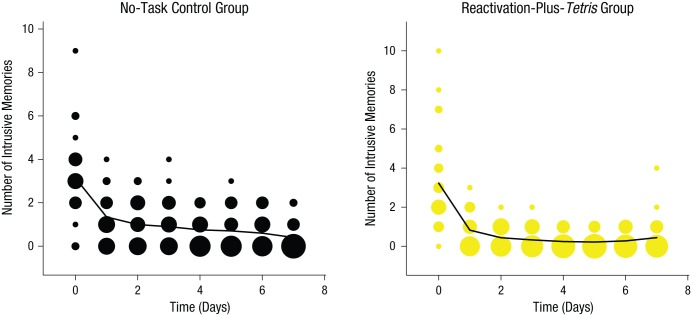
Results from Experiment 1: frequency scatter plots showing the time course of the mean number of intrusive memories reported in the diary daily from Day 0 (prior to intervention) to Day 7, separately for the two groups. Note that the intervention was on Day 1. The solid lines are the results of a generalized additive model (see Equation 1). The size of the bubbles represents the number of participants who reported the indicated number of intrusive memories on that particular day.

**Fig. 3.**
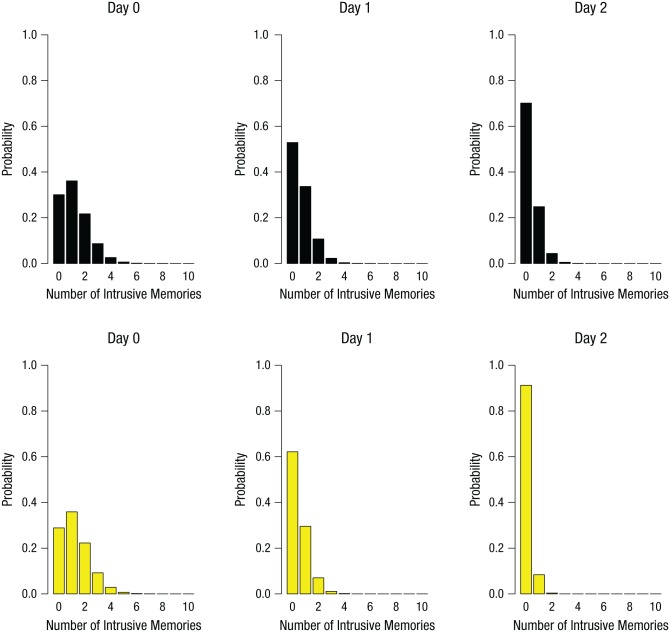
Results of the time-series analysis in Experiment 1: Poisson distribution of the predicted likelihood of intrusive memories in the no-task control group (top row) and the reactivation-plus-*Tetris* group (bottom row), separately for Day 0, Day 1, and Day 2.

To illustrate the trajectory of intrusive memories over time, we conducted a nonlinear time-series analysis using generalized additive models. The number of intrusive memories declined faster in the reactivation-plus-*Tetris* group than in the control group (Fig. 2). Generalized linear models (repeated measures ANCOVA with Poisson errors) in which time was a covariate confirmed a significant interaction effect between experimental group and time, χ^2^(1, *N* = 52) = 8.05, *p* < .01, which illustrated a difference between the time dynamics of intrusive memories between the two experimental groups. As in the time-series analysis, the reactivation-plus-*Tetris* group showed a greater decline in the number of intrusive memories over time than did the control group. Further examination showed that there were no differences in the number of intrusive memories (or predicted distributions) between groups on Days 0 or 1. However, on Day 2 (24 hr after the intervention), the expected probability of no intrusive memories for the reactivation-plus-*Tetris* group (almost completely centered on 100% likelihood of zero intrusive memories) was greater than the expected probability for the control group not having any intrusive memories (Fig. 3). Thus, overall, the group that completed a memory-reactivation task for an experimentally induced aversive memory (24 hr after the event) followed by *Tetris* game play showed a substantially different time course of intrusive memory frequency over the week than did a control group.

#### Recognition memory

As expected on the basis of previous studies, *t* tests showed that there were no differences between groups on tests of either verbal, *t*(50) = 0.93, *p* = .36, or visual, *t*(50) = 1.00, *p* = .32, recognition memory of the film (Table S1 in the Supplemental Material)—this is consistent with the possibility that intrusive-memory frequency (diary/IPT intrusion score) was influenced while leaving recognition memory (for facts and scenes in the trauma film) intact.

### Discussion

There were fewer intrusive memories of the trauma film in the reactivation-plus-*Tetris* group than in the control group. However, the absence of an active control condition limits conclusions, and we do not know the direction of the effects. For example, it could be that in our no-task control group, simply returning to the lab might have strengthened participants’ memory and led to a relative increase in intrusions, whereas there was no effect in our key group. A test of memory reactivation only is warranted. Another possibility is that playing an enjoyable computer game alone could have led to the improvement, and thus *Tetris* alone requires examination. Further, a test of reconsolidation-update mechanisms requires dismantling the intervention—that is, testing its component parts—reactivation and *Tetris*—in isolation.

## Experiment 2

In Experiment 2, we compared four experimental groups. Two of the groups replicated those in Experiment 1: the reactivation-plus-*Tetris* and no-task control groups. Two additional control groups, each consisting of a component part of the key group (*Tetris* only and memory reactivation only), were added to elucidate possible reconsolidation-update mechanisms. Reconsolidation theory (Nader & Hardt, 2009) predicts that old memories are susceptible to disruption only when reactivated and only disrupted if an intervention prevents restabilization (reconsolidation). If playing *Tetris* after memory reactivation reduces intrusion frequency by interfering with memory reconsolidation, then only the combination of film-memory reactivation (to initiate reconsolidation) with *Tetris* game play (to interfere with reconsolidating visual memory for the trauma film) should be effective. Playing *Tetris* alone parallels nonreactivation controls in the reconsolidation literature. Memory reactivation alone should also be insufficient to alter intrusion frequency (Nader & Hardt, 2009). Both components control for nonspecific task effects.

The procedure in Experiment 1 was repeated, with two additions: a *Tetris*-only group played *Tetris* 24 hr after trauma-film viewing without the memory-reactivation task, and a reactivation-only group underwent the memory-reactivation task 24 hr after viewing the film without playing *Tetris*.

### Method

#### Participants

Seventy-two participants (47 females, 25 males; age range = 18–62 years) were recruited using the same methodology as in Experiment 1. Sixty-four percent of participants were students, 26% were employed, 7% were unemployed, and 3% were retired. Participants described their ethnicity as 57% White British, 22% White other, 4% other mixed background, 4% Chinese, 3% mixed White and Black Caribbean, 3% mixed White and Black African, 3% Black African, 1% Mixed White and Asian, 1% Indian, and 1% other Asian background. Ethical approval was obtained from the University of Oxford Central University Research Ethics Committee (reference number: MSD/IDREC/C1/2010/104).

#### Procedure

The first two groups were identical to those in Experiment 1; reactivation-plus-*Tetris* and no-task control (*n* = 18 in each). The third group played *Tetris* only (24 hr after viewing the trauma film, participants played *Tetris* but were not given the memory-reactivation task; *n* = 18). The fourth group experienced reactivation only (24 hr after viewing the trauma film, participants underwent the memory-reactivation task, i.e., viewed 11 still images from the film followed by a 10-min break containing a standardized music filler task, but did not play *Tetris; n* = 18). All other procedures were identical to those in Experiment 1.

#### Statistical analysis

On the basis of the effect size of *d* = 1.14 from Experiment 1, we assumed a large effect size of *f* = 0.4. A sample size of 18 per condition was required in order to ensure an 80% power to detect this difference at the 5% significance level. We continued recruiting until we had reached the number of participants required on the basis of our sample size. Intrusive-memory frequency across the first 24 hr (prior to intervention), score on the Intrusion subscale of the IES-R, recognition memory score, TEQ score, depression, anxiety, age, attention to the film, demand, and diary compliance were analyzed using one-way ANOVAs with experimental condition as a between-groups factor.

One-way ANOVAs followed by planned comparisons were undertaken for intrusive-memory frequency across Days 1 to 7 (equal variances not assumed) and IPT intrusion score. Gender was analyzed between groups using a chi-square test. To assess mood deterioration resulting from viewing the trauma film, we conducted a two-way repeated measures ANOVA with main factors of time (pre- vs. postfilm) and group (reactivation-plus-*Tetris*, no-task control, *Tetris* only, and reactivation only). Two-tailed tests and an alpha level of .05 were used for all statistical comparisons. Time-series analyses were undertaken in *R.* (Analyses of the Intrusion subscale of the IES-R, TEQ score, depression, anxiety, age, attention to the film, diary compliance, gender, and mood deterioration are presented in the Supplemental Material.) To investigate the time-course of intrusions, we used a repeated measures ANCOVA followed by nonlinear time-series analysis, as in Experiment 1.

### Results

Groups were matched at baseline for age and gender, as well as self-report-questionnaire scores for trait anxiety, depression, and trauma history. Mood deterioration during film viewing, postfilm distress, attention to the film, demand ratings, and diary compliance were also matched (see the Supplemental Material).

#### Intrusive memories preintervention

**Fig. 4.**
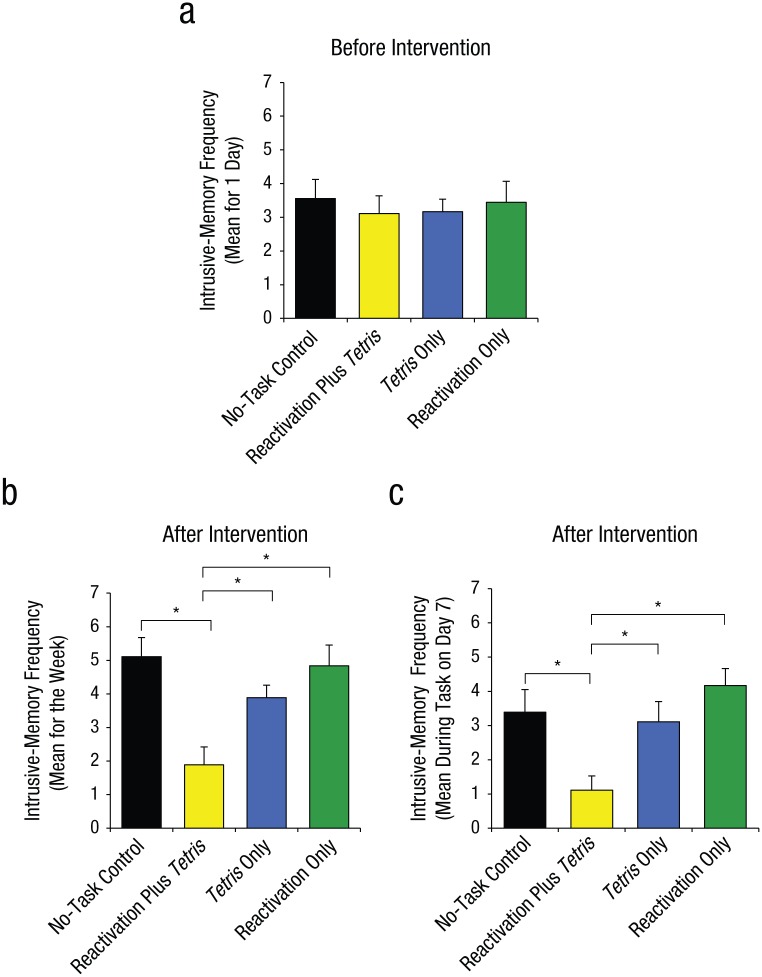
Results from Experiment 2: mean number of intrusive memories recorded in the diary during the first 24 hr following viewing of the experimental trauma film (i.e., preintervention; a), mean number of intrusive memories recorded in the diary across totaled over the 7-day period after the intervention (b), and mean score on the intrusion-provocation task on Day 7 (c). In each graph, results are shown separately for the four groups. Asterisks indicate that results for the reactivation-plus-*Tetris* group were significantly different from results for the other three groups (*p < .01). Error bars represent +1 *SEM*.

First, prior to the intervention (over the first 24 hr after viewing the film: Day 0), we confirmed that the four groups experienced a similar number of intrusive memories, *F*(3, 68) = 0.16, *p* = .92 (Fig. 4a).

#### Intrusive memories postintervention

Second, and critically, for the 7-day diary postintervention, there was a significant difference between groups in overall intrusion frequency in daily life, *F*(3, 68) = 3.80, *p* = .01, η_*p*_^2^ = .14 (Fig. 4b). Planned comparisons demonstrated that relative to the no-task control group, only those in the reactivation-plus-*Tetris* group, *t*(22.63) = 2.99, *p* = .007, *d* = 1.00, experienced significantly fewer intrusive memories; this finding replicated Experiment 1. Critically, as predicted by reconsolidation theory, the reactivation-plus-*Tetris* group had significantly fewer intrusive memories than the *Tetris*-only group, *t*(27.96) = 2.52, *p* = .02, *d* = 0.84, as well as the reactivation-only group, *t*(25.68) = 3.32, *p* = .003, *d* = 1.11. Further, there were no significant differences between the no-task control group and the reactivation-only group, *t*(32.23) = 0.22, *p* = .83, or between the no-task control group and the *Tetris*-only group, *t*(30.03) = 1.01, *p* = .32.

Third, a similar pattern was seen on a convergent measure—the frequency of IPT intrusions on Day 7 in the laboratory, for which there was an overall significant difference between groups, *F*(3, 68) = 5.57, *p* = .002, η_*p*_^2^ = .20 (Fig. 4c; Day 7). Planned comparisons showed that there was a significantly lower intrusion score in the reactivation-plus-*Tetris* group compared with the no-task control group, *t*(68) = 2.92, *p* = .005, *d* = 0.97, which replicated the results of Experiment 1. Critically, in line with reconsolidation theory, the reactivation-plus-*Tetris* group also differed significantly from both the reactivation-only group, *t*(68) = 3.92, *p* < .001, *d* = 1.31, and the *Tetris*-only group, *t*(68) = 2.56, *p* = .01, *d* = 0.85. Further, the reactivation-only group, *t*(68) = 1.00, *p* = .32, and the *Tetris*-only group, *t*(68) = 0.36, *p* = .72, did not differ significantly from the no-task control group.

#### Time course of intrusions

**Fig. 5.**
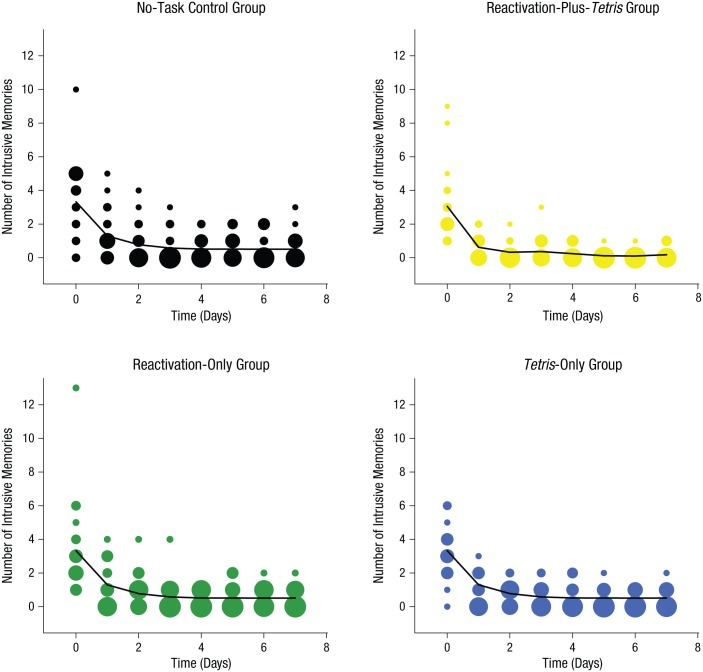
Results from Experiment 2: frequency scatter plots showing the time course of the number of intrusive memories reported in the diary daily from Day 0 (prior to intervention) to Day 7, separately for the four groups. Note that the intervention was on Day 1. The solid lines show the results of a generalized additive model in which the three control groups (no-task control, *Tetris* only, and reactivation only) were fitted with the same line (see Equation 1). The size of the bubbles represents the number of participants who reported the indicated number of intrusive memories on that particular day.

The trajectory of intrusive memories over time declined faster in the reactivation-plus-*Tetris* group than in the other groups (Fig. 5; also see Fig. S2 in the Supplemental Material). Further, generalized linear models (repeated measures ANCOVA with Poisson errors) confirmed no significant difference in the number of intrusive memories over time among the three control groups: no-task control, *Tetris*-only, and reactivation-only groups, χ^2^(4, *N* = 72) = 4.01, *p* = .40; thus, data from these three groups were pooled into a single control group and compared with the reactivation-plus-*Tetris* group. Critically, intrusive-memory frequency over the time course of the experiment for the reactivation-plus-*Tetris* group was significantly different from that in the combined control group, χ^2^(1, *N* = 72) = 15.55, *p* < .01. This illustrates a difference between the time dynamics of intrusive memories between the reactivation-plus-*Tetris* group and the other experimental groups.

**Fig. 6.**
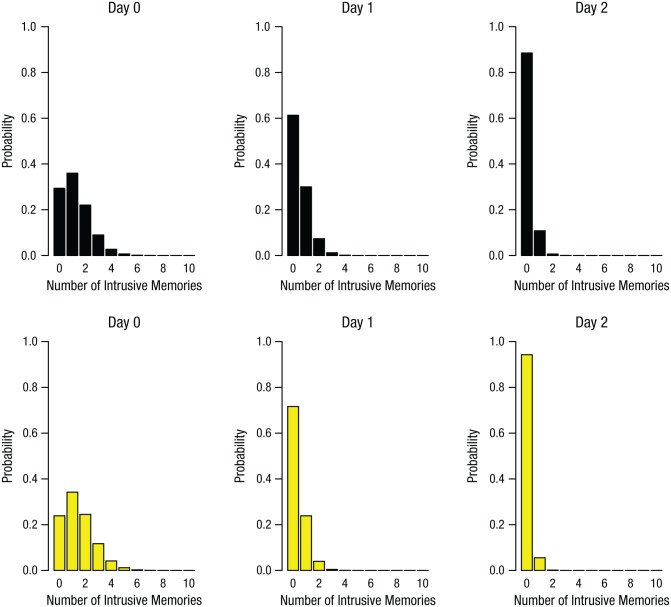
Results of the time-series analysis in Experiment 2: Poisson distribution of the predicted likelihood of intrusive memories in the combined control group (no-task control, *Tetris* only, and reactivation only; top row) and the reactivation-plus-*Tetris* group (bottom row), separately for Day 0, Day 1, and Day 2.

From nonlinear time-series analysis, expected Poisson distributions revealed that by Day 2 (24 hr after intervention), the expected probabilities of no intrusive memories for participants in the reactivation-plus-*Tetris* group (almost completely centered on 100% likelihood of zero intrusive memories) was greater than that predicted for the combined control group (Fig. 6). Thus, a memory-reactivation task for a trauma film (24 hr postfilm) followed by *Tetris* reduced intrusion frequency in the following week if, and only if, game play occurred in combination with the memory reactivation (i.e., consistent with the hypothesis that the combined procedure interfered with intrusive memory reconsolidation).

#### Recognition memory

Finally, as predicted, there was no significant difference among groups on the tests of either visual, *F*(3, 68) = 1.67, *p* = .18, or verbal, *F*(3, 68) = 0.85, *p* = .47, recognition memory of the trauma film (see Table S2 in the Supplemental Material).

## General Discussion

Overall, the results of the present experiments indicate that the frequency of intrusive memories induced by experimental trauma can be reduced by disrupting reconsolidation via a competing cognitive-task procedure, even for established memories (here, events viewed 24 hours previously). That is, a group that performed a task to reactivate an already consolidated memory of a trauma film (to initiate reconsolidation) 24 hr after film exposure, combined with *Tetris* game play, showed substantially fewer intrusions than did a no-task group (Experiments 1 and 2). Critically, neither playing *Tetris* alone (a nonreactivation control condition) nor the control of memory reactivation alone was sufficient to reduce intrusions (Experiment 2). Analogously, simply playing an enjoyable computer game or mere reminders about trauma would be unlikely to reduce intrusions. Rather, their combination is required, which supports a reconsolidation-theory account. We suggest that intrusive-memory reduction is due to engaging in a visuospatial task within the window of memory reconsolidation, which interferes with intrusive image reconsolidation (via competition for shared resources). Results do not permit conclusions about task modality specificity, so future work is therefore warranted.

Our procedure modified intrusion frequency while leaving recognition memory intact, which indicates that trauma-film memory had not been erased but ceased intruding involuntarily. Although consistent with clinical models (Brewin, 2014), the dissociation between intrusions and recognition is contrary to traditional memory models (e.g., Tulving, 2002)—a paradox requiring future research. Perhaps counterintuitively, it is not people’s ability to deliberately remember trauma (episodic memory) but intrusive memories that are the key problem in PTSD. Deliberate recall is important for legal testimony, autobiographical memory, and future safety.

A limitation of this study is that we used a trauma film as an experimental model for trauma and intrusion development. The film content was of events involving actual or threatened death and serious injury (APA, 2013), though this film viewing itself did not meet criteria for a traumatic event. The *DSM–5* allows for exposure to trauma through “electronic media, television, movies or pictures” only if occurring in work-related settings (APA, 2013, p. 271). Our experiments may hold relevance to broader emotional intrusions experienced in daily life (Bernsten, 2010). Another limitation involves only using one computer game—future work should test alternative games hypothesized either to share visuospatial working memory resources with intrusions or to not share such resources (e.g., verbal games). Underlying mechanisms require further examination (e.g., retroactive interference).

From Marcel Proust’s example of sudden childhood recall after eating a madeleine to flashbacks depicted in war films, involuntary memory has long held fascination. The current work bridges a clinical area of public concern (trauma viewing) with animal and human neuroscience. Reconsolidation offers a mechanism through which memory can be modified (strengthened or weakened) and here harnessed to stem the course of emotional intrusions. Understanding cognitive mechanisms underlying intrusive-memory amelioration may help generate more widely available mental-health treatments (Kazdin, 2007). Results also stimulate challenges to traditional models of memory.

This research is the first to investigate the disruption of involuntary memory for emotional events within a reconsolidation framework, using a cognitive procedure. We propose that after memory reactivation, a visuospatial cognitive task (*Tetris*) that competes for the same working memory resources as the reactivated memory (a cognitive blockade) offers a simple noninvasive way to reduce intrusions of a trauma film. A critical next step is to investigate whether findings extend to reducing the psychological impact of real-world emotional events and media. Conversely, could computer gaming be affecting intrusions of everyday events?

## Supplementary Material

Supplementary material

## Supplementary Material

Supplementary material

## Supplementary Material

Supplementary material

## Supplementary Material

Supplementary material

## Supplementary Material

Supplementary material