# Supplementary material for: Computer Game Play Reduces Intrusive Memories of Experimental Trauma via Reconsolidation-Update Mechanisms
Source: Psychol Sci. 2015 Aug;26(8):1201–15. doi: 10.1177/0956797615583071 (PMC4526368; doi:10.1177/0956797615583071)
Supplement: Supplementary material [file DS_10.11770956797615583071_TableS2.pdf]

DOI: 10.1177/0956797615583071

Table S2

Experiment 2: Baseline characteristics, manipulation checks and recognition memory scores

	No-Task Control (n = 18)	Reactivation+ Tetris (n = 18)	Tetris-Only (n = 18)	Reactivation-Only (n = 18)
	n (%)	*n* (%)	*n* (%)	*n* (%)
Female	14 (77.78%)	9 (50.00%)	14 (77.78%)	10 (55.56%)
	M [95% CI]	*M* [95% CI]	*M* [95% CI]	*M* [95% CI]
Age	23.67 [20.28, 27.06]	25.44 [21.77, 29.12]	25.39 [21.50, 29.28]	28.39 [22.77, 34.01]
Number of traumatic events	1.22 [0.33, 2.12]	0.78 [0.31, 1.25]	0.94 [0.45, 1.44]	1.00 [0.58, 1.42]
Beck Depression Inventory-II	3.33 [1.06, 5.61]	2.67 [1.45, 3.88]	3.61 [1.33, 5.89]	2.11 [0.73, 3.50]
State-Trait Anxiety Inventory	33.39 [28.25, 38.53]	34.67 [30.92, 38.41]	33.94 [29.67, 38.22]	33.78 [29.77, 37.79]
Attention to film	9.67 [9.43, 9.91]	9.28 [8.87, 9.69]	9.56 [9.25, 9.86]	9.44 [9.14, 9.75]
Diary compliance	8.56 [8.13, 8.98]	8.17 [7.26, 9.07]	8.44 [7.93, 8.96]	8.44 [7.99, 8.90]
Mood rating pre-film	3.30 [2.24, 4.36]	4.12 [2.64, 5.60]	3.93 [2.52, 5.33]	4.50 [2.88, 6.12]
Mood rating post-film	15.00 [11.07, 18.93]	15.78 [12.69, 18.87]	17.72 [12.89, 22.56]	15.85 [12.24, 19.46]
Post-film distress	6.44 [5.51, 7.38]	6.44 [5.61, 7.28]	6.11 [4.82, 7.40]	6.06 [4.82, 7.29]
Visual recognition memory	15.00 [13.96, 16.04]	15.67 [14.59, 16.75]	14.28 [13.35, 15.21]	14.44 [13.40, 15.49]
Verbal recognition memory	19.78 [18.65, 20.90]	18.33 [17.41, 19.25]	18.83 [17.28, 20.39]	18.89 [17.15, 20.63]

DOI: 10.1177/0956797615583071

Demand rating	-1.33<br>[-2.79, 0.12]	-1.78<br>[-3.67, 0.11]	-1.61<br>[-3.24, 0.02]	-0.28<br>[-2.16, 1.60]

Note. A composite mood score pre- and post-film was a calculated by summing participants ratings on six visual analogue scales; fear, horror, anxiety, sadness, hopelessness and depressed. Visual and verbal recognition memory scores are a sum of correct responses (out of a maximum score of 22 for visual and 32 for verbal recognition memory tests).
