# Supplementary material for: Computer Game Play Reduces Intrusive Memories of Experimental Trauma via Reconsolidation-Update Mechanisms
Source: Psychol Sci. 2015 Aug;26(8):1201–15. doi: 10.1177/0956797615583071 (PMC4526368; doi:10.1177/0956797615583071)
Supplement: Supplementary material [file DS_10.11770956797615583071_TableS1.pdf]

DOI: 10.1177/0956797615583071

Table S1

Experiment 1: Baseline characteristics, manipulation checks and recognition memory scores

	No-Task Control (n = 26)	Reactivation+ Tetris (n = 26)
	n (%)	*n* (%)
Female	18 (69.23)	13 (50.00)
	M [95% CI]	*M* [95% CI]
Age	24.85 [22.80, 26.89]	24.08 [21.25, 26.89]
Number of traumatic events	0.96 [0.47, 1.45]	1.19 [0.70, 1.68]
Beck Depression Inventory-II	4.54 [2.66, 6.42]	3.38 [2.21, 4.56]
State-Trait Anxiety Inventory	35.04 [31.94, 38.14]	33.50 [30.68, 36.32]
Attention to film	9.15 [8.72, 9.59]	9.12 [8.70, 9.53]
Diary compliance	8.23 [7.81, 8.65]	8.31 [7.53, 9.08]
Mood rating pre-film	5.66 [3.33, 7.99]	3.86 [2.56, 5.15]
Mood rating post-film	18.84 [14.18, 23.49]	14.49 [10.78, 18.19]
Post-film distress	6.50 [5.66, 7.34]	5.81 [4.72, 6.90]
Visual recognition memory	14.27 [13.12, 15.42]	15.00 [14.02, 15.98]
Verbal recognition memory	19.38 [18.10, 20.67]	18.58 [17.34, 19.81]
Demand rating	- 0.23 [-1.43, 0.96]	-1.27 [-2.50, -0.04]

Note. A composite mood score pre- and post-film was a calculated by summing participants ratings on six visual analogue scales; fear, horror, anxiety, sadness, hopelessness and depressed. Visual and verbal recognition memory scores are a sum of correct responses (out of a maximum score of 22 for visual and 32 for verbal recognition memory tests).
