# Supplementary material for: Computer Game Play Reduces Intrusive Memories of Experimental Trauma via Reconsolidation-Update Mechanisms
Source: Psychol Sci. 2015 Aug;26(8):1201–15. doi: 10.1177/0956797615583071 (PMC4526368; doi:10.1177/0956797615583071)
Supplement: Supplementary material [file DS_10.11770956797615583071_Supplemental_Material.pdf]

DOI: 10.1177/0956797615583071

Computer game-play reduces intrusive memories of experimental trauma via reconsolidation update mechanisms

Ella L. James, Michael B. Bonsall, Laura Hoppitt, Elizabeth M. Tunbridge, John R. Geddes, Amy L. Milton, & Emily A. Holmes

Supplemental Material

Experiment 1: Additional procedures

After the experiment, all participants' diary descriptions of the content of their image-based intrusions (e.g. "*image of boy knocked down while crossing the road texting*" and "*I saw a man struggling to stay above the surface of the sea*") were matched to the one scene in the film where this event occurred. Accordingly, 94% of the intrusion descriptions matched to the film, and were included in subsequent data analyses. A second independent rater judged a random sample of diaries (n = 15 diaries; 29 %). An inter-rater reliability analysis using the k statistic was $k = 0.84$, $p < .001$, suggesting an excellent level of reliability for matching diary intrusion descriptions to film sequences and overall that the intrusions reported were indeed of the film (rather than other events).

At the end of session three participants completed a 'demand rating' in which they were asked to rate how much they thought that performing *Tetris* after a distressing film would increase or decrease intrusive memories of the film using a 21 point linear scale from -10 '*extremely decrease*', through 0 '*no effect*', to +10 '*extremely increase*'.

Experiment 1: Additional methods

Self-rated Tetris performance*:* Immediately following *Tetris* game-play, participants were asked 'How difficult or easy did you find the game you just played?' and marked their rating on a 10cm line from '*Not difficult at all/Easy*' to '*Extremely Difficult/Hard.*

Tetris Game Score: The *Tetris* game-play score generated by the computer program was noted. The game (Tetris Zone, 2007) records a score mainly derived from the number of lines that have been created and the Level that the player has reached. The blocks fall at a greater pace as the game progresses, and the points awarded per horizontal line completed is a multiple of the Level number. Thus, the potential to score points increases as the player progresses through the Levels. Further, participants can enhance their score by performing various 'tricks' (e.g. spinning a block as opposed to simply letting it fall into place). The game is adaptive, and therefore tailored for individual ability, thus only the better players will reach the levels that afford the highest points. The cumulative total score was analysed.

Experiment 1: Supplemental results

A chi-squared test confirmed that the time of day of session one did not differ between groups, $p > .3$. There were no significant differences between groups for prior trauma history, $t\ (50) = 0.66$, $p = .51$, depression, $t\ (50) = 1.07$, $p = .29$, anxiety, $t\ (50) = 0.76$, $p = .45$, age, $t\ (50) = 0.46$, $p = .65$, post-film distress, $t\ (50) = 1.04$, $p = .31$, attention to the film, $t\ (50) = 0.13$, $p = .90$, diary compliance, $t\ (50) = 0.18$, $p = .86$, or gender, $\chi^2\ (1, N = 52) = 2.00$, $p = .16$, see Table S1.

To verify that mood scores deteriorated after viewing the trauma film, but that this deterioration was not different between groups (i.e. no time x group interaction) we used a two-way repeated measures analysis of variance (ANOVA) with time (pre- to post-film) and group (reactivation-plus-*Tetris* and no-task control) as factors. This analysis confirmed that mood deteriorated for both groups from pre- to post-film, Time: $F\ (1, 50) = 95.59$, $p < .001$ $n_p^2 = 0.66$, and that there was no difference between experimental groups (Time x Group: $F\ (1, 50) = 1.09$, $p = .30$); Table S1.

DOI: 10.1177/0956797615583071

The reactivation-plus-*Tetris* group had significantly lower scores on the Intrusion subscale of the IES-R (M = .68, SD = .53) on Day 7 as compared to the no-task control group (M = 1.01, SD = .41), t (50) = 2.50, p = .016, d = 0.69.

Overall, mean demand ratings were close to 0 (M = -0.75, SD = 3.02) and importantly showed no significant differences between groups, t (50) = 1.25, p =.22, see Table S1. There was no relationship between demand ratings and intrusive memory frequency for either the no-task control group (r = -.16, p = .42) or the reactivation –plus-*Tetris* group (r = -.15, p = .48). These data suggest that participants were not aware of the purpose of the experiment and that demand characteristics are unlikely to be affecting the results.

To explore whether performance on *Tetris* was related to the decline in intrusions, self-rated Tetris task performance and computerised *Tetris* Game-Play Score were examined in relation to frequency of intrusive memories in the diary. The analysis (as Day 1-7 intrusions are total number of counts) used a Poisson regression with a square root transformation of the explanatory variable (Self-rated *Tetris* performance M = 3.20, SD = 2.67; and *Tetris* Game-Play Score M = 30396.50; SD = 22124.74). The slopes of both lines are significantly different from zero, in the predicted directions. Thus, increasing self-rated task difficulty was positively associated with a higher frequency of intrusions (slope = 0.441 [SE = 0.158], p = .0053), see Fig. S1a. Higher game-play scores (reflecting task success) were associated with fewer intrusions (slope = - 0.004 [SE = 0.0017], p = .020), see Fig. S1b.

DOI: 10.1177/0956797615583071

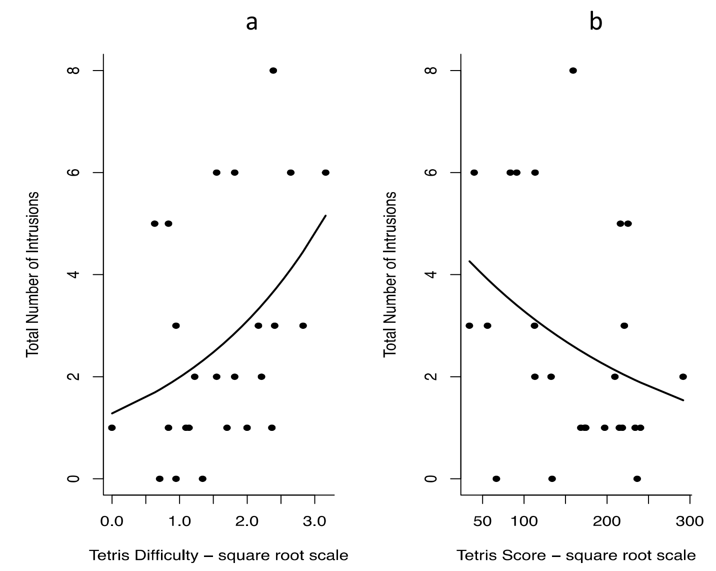

Fig. S1. Within the reactivation-plus-*Tetris* group of Experiment 1, the number of intrusive memories over days 1-7 is significantly associated with both indices of *Tetris* performance (self-rated *Tetris* performance and Tetris Game-Play Score).

Experiment 2: Additional procedures

As in Experiment 1, after the experiment, all participants' diary descriptions of the content of their image-based intrusions were matched to the one scene in the film where this event occurred. Ninety-five percent of the diary intrusion descriptions matched to the film. Independent inter-rater agreement on a random sample of participants' diaries (n = 21; 29 %) again indicated excellent consensus; k = 0.79; p < .001.

As in Experiment 1, at the end of session three participants completed a 'demand rating' in which they were asked to rate how much they thought that performing *Tetris* after a distressing film would increase or decrease intrusive memories of the film using a 21 point linear scale from -10 '*extremely decrease*', through 0 '*no effect*', to +10 '*extremely increase*'.

Experiment 2: Supplemental results

A chi-squared test confirmed that the time of day of session one did not differ between the four experimental groups, $p > .3$. There were no significant differences between the four experimental groups for prior trauma history, F (3, 68) = 0.42, p = .74, depression, F (3, 68) = 0.59, p = .62, anxiety, F (3, 68) = 0.07, p =.98, age, F (3, 68) = 0.95, p =.42, post-film distress, F (3, 68) = 0.16, p = .92, attention to the film, F (3, 68) = 1.18, p = .32, diary compliance, F (3, 68) = 0.33, p = .80, gender, χ^2 (3, N = 72) = 5.09, p = .17, or age F (3, 68) = 0.95, p = .42, see Table S2.

To again verify that mood scores deteriorated over time as a result of watching the trauma film, but that this deterioration was not different between the groups, we used a two-way repeated measures (ANOVA) with time (pre- to post-film) and group (reactivation-plus-*Tetris*, no-task control, *Tetris*-only and reactivation-only) as factors. The analysis confirmed that mood deteriorated for both groups from pre- to post-film, Time: F (1, 68) = 206.84, p < .001, n_p^2 = 0.75, with no differences between groups (Time x Group: F (3, 68) = 0.44, p = .72); Table S2.

No significant difference was found between the four groups on the IES-R for the Intrusion Subscale (reactivation-plus-*Tetris*, M = 0.61, SD = 0.38; no-task control M = 0.81, SD = 0.58; *Tetris*-only, M = 0.74, SD = 0.50; reactivation-only, M = 0.81, SD = 0.38), F (3, 68) = 0.73, p = .54.

Overall, mean demand ratings were close to 0 (M = -1.25, SD = 3.44) and importantly showed no significant differences between conditions, F < 1, see Table S2. As before there was no relationship between demand ratings and intrusive memory frequency for any group: no-task control (r = .10, p = .69); reactivation-plus-*Tetris* (r = .07, p = .79); *Tetris*-only (r = -0.19, p = .44) and reactivation-only (r = -0.17, p = .50). These data suggest that participants were not aware of the purpose of the experiment and that demand characteristics are unlikely to be affecting the results.

DOI: 10.1177/0956797615583071

We again examined any relationship between diary intrusions (days 1-7) and *Tetris* performance (self-rated *Tetris* performance $M = 2.96$, $SD = 1.69$; and *Tetris* Game-Play Score $M = 31843.61$, $SD = 27990.23$) in the reactivation-plus-*Tetris* group ($N = 18$). There was no significant association between either score with intrusions (self-rated task performance difficulty: slope = 0.5758 [$SE = 0.3551$], $p = .105$; game score slope = -0.0030 [$SE = 0.002249$], $p = .181$).
